# Caffeine Functions by Inhibiting Dorsal and Ventral Hippocampal Adenosine 2A Receptors to Modulate Memory and Anxiety, Respectively

**DOI:** 10.3389/fphar.2022.807330

**Published:** 2022-02-02

**Authors:** Yawei Xu, Yalei Ning, Yan Zhao, Yan Peng, Fen Luo, Yuanguo Zhou, Ping Li

**Affiliations:** State Key Laboratory of Trauma, Department of Army Occupational Disease, The Molecular Biology Center, Burn and Combined Injury, Daping Hospital, Army Medical University (Third Military Medical University), Chongqing, China

**Keywords:** caffeine, dorsal hippocampus, ventral hippocampus, adenosine A2A receptor, memory, anxiety

## Abstract

As a nonspecific antagonist of the adenosine A_2A_ receptor (A_2A_R), caffeine enhances learning and improves memory impairment. Simultaneously, the consumption of caffeine correlates with a feeling of anxiety. The hippocampus is functionally differentiated along its dorsal/ventral axis and plays a crucial role both in memory and anxiety. Whether caffeine exerts its regulation by inhibiting A_2A_Rs in different subregions of the hippocampus is still unknown. In the present study, we found that after chronic intake of drinking water containing caffeine (1 g/L, 3 weeks), mice exhibited aggravated anxiety-like behavior and enhanced memory function. Tissue-specific, functional disruption of dorsal hippocampal A_2A_Rs by the CRE-LoxP system prevented the memory-enhancing effect of caffeine, while selective disruption of ventral hippocampal A_2A_Rs blocked the impact of caffeine on anxiety. These results, together with the enhanced memory of dorsal hippocampus A_2A_R knockout mice and greater anxiety-like behavior of ventral hippocampus A_2A_R knockout mice without caffeine, indicates a dissociation between the roles of ventral and dorsal hippocampal A_2A_ receptors in caffeine’s effects on anxiety-like and memory-related behavioral measures, respectively. Furthermore, optogenetic activation of dorsal or ventral hippocampal A_2A_Rs reversed the behavioral alterations caused by drinking caffeine, leading to impaired memory or decreased anxiety-like behaviors, respectively. Taken together, our findings suggest that the memory- and anxiety-enhancing effects of caffeine are related to the differential effects of inhibiting A_2A_Rs in the dorsal and ventral hippocampus, respectively.

## Introduction

As the most widely consumed psychotropic substance and a component of the most popular beverages, caffeine is used to counteract performance impairments associated with sleep loss (Irwin et al., 2020).The consumption of caffeine attenuates memory impairments associated with aging and Alzheimer’s disease (AD) (Solfrizzi et al., 2015; Jacobson et al., 2020; Londzin, et al., 2021)and enhances memory in healthy humans (Borota et al., 2014; Irwin et al., 2020). However, controlled studies have confirmed that caffeine produces negative effects such as increased anxiety (Garcia and Salloum, 2015; Khurana and Bansal, 2019). Similarly, mouse studies have also shown that caffeine ingestion improves memory and induces anxiety (Xu and Reichelt, 2018). The main molecular targets of caffeine in the brain are adenosine receptors, including the inhibitory A_1_ receptor (A_1_R) and facilitatory A_2A_ receptor (A_2A_R) (Ikram et al., 2020). From results obtained in animal models, it was further concluded that the effect of caffeine on both anxiety and memory performance was mimicked by the selective blockade of A2A, but not of A1 receptor (Kaster et al., 2015; Machado et al., 2017).

The hippocampus belongs to the limbic system and plays an important role in memory, spatial navigation and emotion. The hippocampus can be divided into two segments along its longitudinal axis: the dorsal and ventral subregions. Early anatomical studies demonstrated differences between the afferent and efferent nerve projections of the dorsal hippocampus (dHPC) and ventral hippocampus (vHPC) (Swanson and Cowan, 1977). According to a current consensus, the role played by the most dorsally located hippocampal segment is on cognitive operations like spatial navigation, while internally monitoring functions related to emotionality are taken on by the ventral segment of the hippocampus (Trompoukis and Papatheodoropoulos, 2020). Moreover, some studies have found that caffeine reverts memory impairment in a depression-prone mouse strain with upregulation of adenosine A_2A_ receptors in the hippocampus (Machado et al., 2017), while selective A_2A_R knockout in the forebrain region (striatum, hippocampus, and cortex) induces anxiety-like behavior (Wei et al., 2014). All the evidence suggests that the hippocampal adenosine A_2A_ receptor is likely to emerge as an important receptor in the regulation of caffeine on memory and anxiety. However, whether the different effects of caffeine on overall function are derived from its action on adenosine A_2A_ receptors in different subregions of the hippocampus is still unclear.

To explore the above question, we first verified that overall A_2A_R knockout reduced the effect of caffeine on mice. Furthermore, we found that localized knockout of dHPC A_2A_Rs reduced the regulation of caffeine on memory but did not affect anxiety, whereas localized knockout of vHPC A_2A_Rs reduced the regulation of caffeine on anxiety without affecting memory. To confirm the memory-enhancing effects of dHPC A2AR knockout, we assessed the level of SNAP-25, a synaptic marker reflecting synapse formation and remodeling (Batista et al., 2017). To confirm the anxiogenic effects of vHPC A2AR knockout, we assessed the level of SNAP-25, a glutamatergic-selective marker and labels excitatory glutamatergic neurons (Zheng et al., 2015; Martineau et al., 2017). Finally, specific stimulation of dorsal/ventral hippocampal A_2A_Rs by optogenetic techniques demonstrated that dorsal hippocampal A_2A_R activation impaired memory while ventral hippocampal A_2A_R activation reduced anxiety. Therefore, our experiments showed that caffeine regulates memory and anxiety by inhibiting the dorsal and ventral hippocampal A_2A_Rs, respectively.

## Materials and Methods

### Animals

Adult male C57BL/6 mice (weighing 25–30 g, 11–13 weeks old) were purchased and used in our study. Global A_2A_R knockout mice were established on a C57BL/6 background as our previously described (Zeng et al., 2020), and their littermates were used as wild-type (WT) mice in this experiment. Mice with a ‘floxed’ adenosine A_2A_R gene (A_2A_^flox/flox^ mice) created by insertion of loxP sequences into introns flanking an exon of the A_2A_R gene (Bastia et al., 2005; Yu et al., 2009) were provided by Dr. Chen. The experimental procedures were performed in accordance with the guidelines of the Animal Ethical and Welfare Committee of the Army Medical University.

### Viral Production

For conditional knockout (Cre/loxp system) of neuronal A_2A_R, pAAV-hSyn-EGFP-2A-CRE virus and pAAV-hSyn-MCS-EGFP-3Flag virus (control virus) were packaged and supplied by OBiO Technology (Shanghai) Corp. Ltd.

For optogenetic manipulations of neuronal A_2A_R, a chimeric rhodopsin-A_2A_R protein (optoA_2A_R) was developed by replacing the intracellular domain of rhodopsin with that of A_2A_R as described in our previous research (Li et al., 2015). Extracellular adenosine or caffeine cannot react with optoA_2A_R. Similarly, pAAV-CaMKIIa-optoA_2A_R-mCherry virus and pAAV-CaMKIIa-MCS-mCherry-3FLAG virus (control virus) were constructed and supplied by OBiO Technology (Shanghai) Corp. Ltd. All viruses were used at titers of ∼4–8*10^12^ vg/ml.

### Drug Treatments

In the first part of our experiment, mice (knockout or wild-type) were randomly allocated to two groups: a control group provided with drinking water without drug and a treatment group provided with drinking water containing caffeine (1 g/L, Sigma) starting 3 weeks before behavioral tests. This dose and schedule of administration of caffeine was chosen since it was sufficient to improve memory and increase anxiety according to a previous study (Kaster et al., 2015).

In the second part of the study, A_2A_^flox/flox^ mice were injected with CRE virus or EGFP virus. Half the mice in each group were then provided with caffeine-containing drinking water starting 3 weeks before behavioral tests, and the other half were provided with normal drinking water.

In the last part, wild-type mice were randomized into two groups: an experimental group injected with the optoA_2A_R virus and a control group were injected with the mCherry virus. Then, all mice underwent optical fiber implantation and were treated with caffeine starting 3 weeks before optogenetic manipulation.

In all experiments, the drinking water with or without caffeine were provided every day, until the mice were killed.

### Animal Surgery: Virus Injection and Optical Fiber Implantation

For all surgical procedures, mice were anesthetized with 1.5% isoflurane at an oxygen flow rate of 1 L/min and then immobilized in a Robot Stereotaxic apparatus (Neurostar, Tübingen, Germany). The fur was shaved, and a midline scalp incision was made for surgical procedures. After surgery, the mice were given saline containing buprenorphine (0.13 mg/kg, subcutaneously) for 3 days for analgesia.

For brain region-specific knockout of A_2A_R, 1.5 μL pAAV-hSyn-EGFP-2A-CRE virus (or control virus) was injected into the each dHPC (AP: 1.1 mm; ML: ±1.25 mm; DV: +1.75 mm) or each vHPC (AP: 3.4 mm; ML: ±2.5 mm; DV: +4.0 mm) of A_2A_^flox/flox^ mice (Total 3.0 μL).

To express optoA_2A_R in hippocampal neurons, we injected 1.5 μL pAAV-CaMKIIa-OptoA_2A_ (A400S)-mCherry virus (or control virus) into the left dorsal or ventral subregion (viral injection coordinates are described above). Four weeks after injection, a 200 μm optical fiber (Shanghai Fiblaser Technology Co., Ltd.) was implanted in the same coordinates. Via a patch cable, the optical fiber was connected to a 473 nm DPSS laser (100 mW; Shanghai Laser and Optics Century). The power density at the fiber tip was approximately 5 mW/mm^2^, and light was delivered with a 50 m pulse width (10Hz). For the optogenetic experiment, mice were habituated with an optical fiber connected to the optical patch cable without laser stimulation for 30 min before the behavior tests, and optical stimulation was delivered specifically according to the different behavioral tests (see below). For immunofluorescence analyses, mice were euthanized by cervical dislocation following 10 min of optical stimulation.

### Behavioral Experiments

To eliminate the acute effects of caffeine while preserving its long-term chronic effects, all behavioral tests were performed within 24–48 h after 3-week caffeine treatment.

Open Field Test: Mice were placed in a square chamber (40 cm × 40 cm × 40 cm length-width-height) with a dimly lit (approximately 65 lux). The center zone was a 20 × 20 cm square. Mice were allowed to freely explore the environment for 5 min, while activities were recorded and analyzed by EthoVision XT behavioral tracking software (Noldus Information Technology Inc.). For optogenetic manipulation, light stimulation was maintained for the entire 5-min duration of the open-field test.

Elevated Plus Maze (EPM) Test: The elevated plus maze had open and closed arms (30 cm × 8.5 cm length-width, 20 cm tall closed arms) that extended from a central platform (8.5 × 8.5 cm). The maze was placed at a height of 58 cm and the room light was about 65 lux. The mouse was placed in the central platform of the maze facing an open arm and were allowed to freely explore the EPM for 5 min, and their activities were recorded and analyzed by EthoVision software (Noldus Information Technology Inc.). For optogenetic experiments, light stimulation was used for a total period of 5 min while mice explored the EPM.

Novel Object Recognition (NOR) **Test:** NOR test was carried out in a home-cage arena (45 cm × 45 cm × 50 cm length-width-height) which they were allowed to freely explore for 10 min on the previous day. In the training trial on the next day, mice were presented with two same objects (a red cube, 2.8 cm × 2.8 cm × 2.8 cm) placed in opposite corners for 10 min. The exploration of the objects, defined by mice showing investigative behaviors (head orientation or sniffing) or playing within 1 cm around the object, was measured. In the testing trial (24 h later), one of the identical objects was changed for a novel object (a yellow pyramid, height 3.0 cm, base 2.8 cm × 2.8 cm), and the animals were left in the cage for 10 min. The exploration time for the familiar and the novel object during the test phase was recorded with EthoVision software. For the optogenetic experiment, light stimuli were only delivered during the testing trial.

Y-Maze Test: Y-maze test was carried out in a gray maze formed by three arms (28 cm × 8.5 cm × 20 cm length-width-height, 58 cm above the ground) so as to form a Y shape. Each mouse was first allowed to explore the maze for 5 min while one arm was blocked (acquisition phase). After 2 h, mice had access to all three arms for a 5 min period (retrieval phase). During the second period, the time spent in each arm was measured by a video-tracking system (Noldus Information Technology Inc.). For the optogenetic experiment, light stimuli were only presented during the retrieval trial.

### Immunofluorescence

Following behavioral experiments or light stimulation, mice were sequentially perfused with saline and 4% paraformaldehyde in PBS. Brains were postfixed, and coronal sections (30 μm) were cut and prepared for immunofluorescence. Free-floating sections were washed in PBS and then incubated for 30 min in 0.3% Triton X-100 and 3% BSA or goat serum. Sections were incubated with the following primary antibodies overnight at 4°C: anti-A_2A_R (1:200, Frontier Institute, AB_2571655), anti-c-Fos (1:50, Santa Cruz, sc-271243), anti-synaptosomal-associated protein 25 (SNAP-25; 1:100, Abcam, ab5666) and anti-vesicular glutamate transporter 1 (vGluT1; 1:100, Abcam, ab227805). Sections were then washed with PBS and incubated with fluorescence-tagged secondary antibodies including Cy3 (1:500, donkey anti-goat, Abcam, ab6949), Cy3 (1:500, goat anti-mouse, Abcam, ab97035), Cy3 (1:500, goat anti-rabbit, Abcam, ab6939), and Alexa Fluor 488 (1:500, goat anti-mouse, Abcam, ab150117) for 1 h at 37°C. Nuclei were subsequently stained with DAPI (Santa Cruz, sc-359850). High-magnification images were captured using a confocal laser-scanning microscope (Leica TCS-SP2, laser lines at 488, 543, 633) and analyzed with Image-Pro Plus 4.5 software. For quantification of immunofluorescence, at least 50 cells were evaluated in each field (3 fields per slice, three slices per mouse, three mice from each analyzed group).

### Statistical Analysis

Results are expressed as the means ± SEM. All semi-quantitative assessments of histological staining were made by a single investigator blinded to the genotype and treatment of the experimental animals. Sample size was chosen according to previous reports and our pre-experiments. Two-way analyses of variance (ANOVAs) were used to assess the effects of caffeine, gene manipulation and the caffeine × gene manipulation interaction in the A_2A_R total (or region-specific) knockout mice. Two-way ANOVAs were used to assess the effects of optoA_2A_R virus, light stimulation and the optoA_2A_R virus × light stimulation interaction in the optoA_2A_R mice. A value of *p* < .05 was considered statistically significant.

## Results

### Global A_2A_R Knockout Blocked the Effect of Caffeine on Anxiety and Memory

**FIGURE 1:**
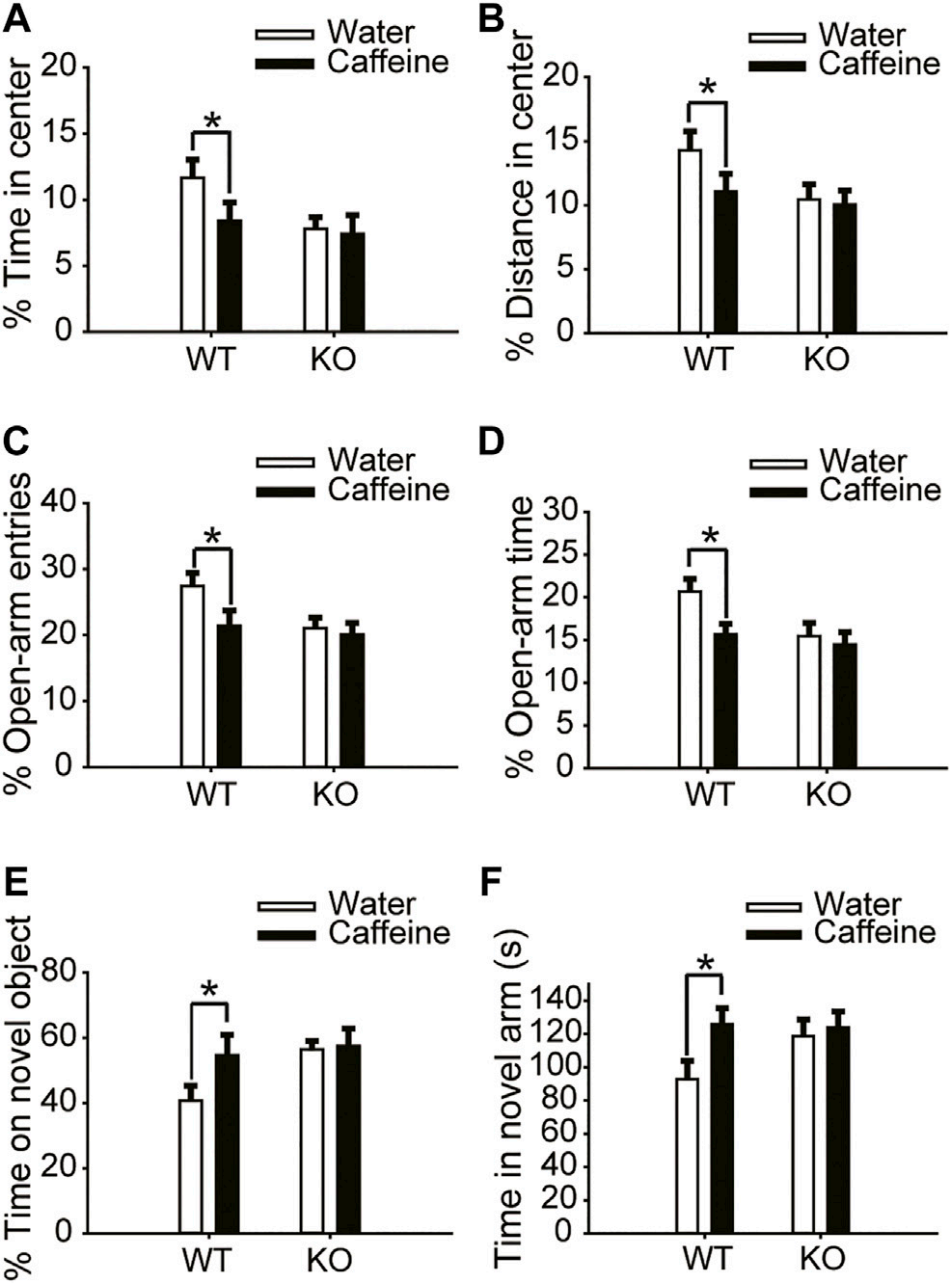
Effects of chronic caffeine consumption on wild-type mice and A_2A_R knockout mice in behavioral experiments. **(A,B)** Behavior of the four groups of mice in the open-field test. Caffeine induced anxiogenic behavior with decreased time in the center **(A)** and distance in the center **(B)** in wild-type mice. **(C,D)** Behavior of the four groups of mice in the Elevated Plus Maze (EPM) test. Caffeine decreased the percentage of open-arm entries **(C)** and the percentage of time spent in the open arms **(D)** in wild-type mice. **(E)** Behavior of the four groups of mice in the Novel Object Recognition (NOR) test. The time spent exploring a novel object is shown as the ratio of the total time spent exploring both objects in the testing trial. **(F)** Behavior of the four groups of mice in the Y-maze test. The time spent in the novel arm is shown during the retrieval phase. Data are presented as the mean ± SEM, *n* = 7 mice per group; *p < .05, two-way ANOVA, Bonferroni post hoc t-test.

The open-field test and EPM test were used to evaluate anxiety-like behaviors. Chronic caffeine consumption (3 weeks) significantly reduced the time (Figure 1A, for caffeine, F_(1,28)_ = 5.741, *p* < .01; for knockout, F_(1,28)_ = 16.341, *p* < .01; for caffeine × knockout interaction, F_(3,28)_ = 4.124, *p* < .01) and distance (Figure 1B, for caffeine, F_(1,28)_ = 56.114, *p* < .001; for knockout, F_(1,28)_ = 23.554, *p* < .01; for caffeine × knockout interaction, F_(3,28)_ = 4.451, *p* < .01) in the center of the open field, and had no significant effect on the total distance (Supplementary Figure S1A, for caffeine, F_(1,28)_ = 0.372, *p* = .547; for knockout, F_(1,28)_ = .001, *p* = .975; for caffeine × knockout interaction, F_(3,28)_ = 0.985, *p* = 0.331). In the EPM, caffeine treatment decreased the percentage of time (Figure 1D, for caffeine, F_(1,28)_ = 9.125, *p* < .01; for knockout, F_(1,28)_ = 21.934, *p* < .01; for caffeine × knockout interaction, F_(3,28)_ = 23.344, *p* < .01) and number of entries (Figure 1C, for caffeine, F_(1,28)_ = 14.624, *p* < .01; for knockout, F_(1,28)_ = 21.679, *p* < .01; for caffeine × knockout interaction F_(3,28)_ = 8.824, *p* < .01) in the open arms which confirmed the open-field test results. We next examined object recognition to evaluate the role of caffeine in memory formation. The caffeine-treated mice showed greater exploration of the novel object over the familiar object (Figure 1E, for caffeine, F_(1,28)_ = 12.347, *p* < .01; for knockout, F_(1,28)_ = 15.472, *p* < .01; for caffeine × knockout interaction, F_(3,28)_ = 14.147, *p* < .01). The spatial recognition memory of mice was probed using a two-trial Y-maze paradigm. Likewise, caffeine-treated mice showed increased time in the novel arm compared with control mice (Figure 1F, for caffeine, F_(1,28)_ = 22.712, *p* < .01; for knockout, F_(1,28)_ = 15.755, *p* < .01; for caffeine × knockout interaction, F_(3,28)_ = 21.457, *p* < .01). This finding suggests that chronic caffeine consumption for 3 weeks enhanced memory performance in mice.

However, chronic caffeine consumption did not alter anxiety-like behaviors in global A_2A_R knockout mice. There were no significant differences between caffeine-treated mice (caffeine + knockout) and control mice (water + knockout) in either the open-field test or the EPM test (Figure 1C and Figure 1D). At the same time, A_2A_R knockout blocked the effect of caffeine in the NOR test and Y-maze test (Figures 1E,F), suggesting that the effect of caffeine on memory was prohibited by A_2A_R inactivation. It is worth noting that in global A_2A_R knockout mice, there were baseline differences in behaviors that are independent of caffeine (Figures 1A–F). But the costimulation with caffeine and A_2A_R knockout did not produce additive effects which reflected the interaction between caffeine-A_2A_R function.

### Dorsal Hippocampal A_2A_R Knockout Blocked the Caffeine-Induced Enhancement of Memory

**FIGURE 2:**
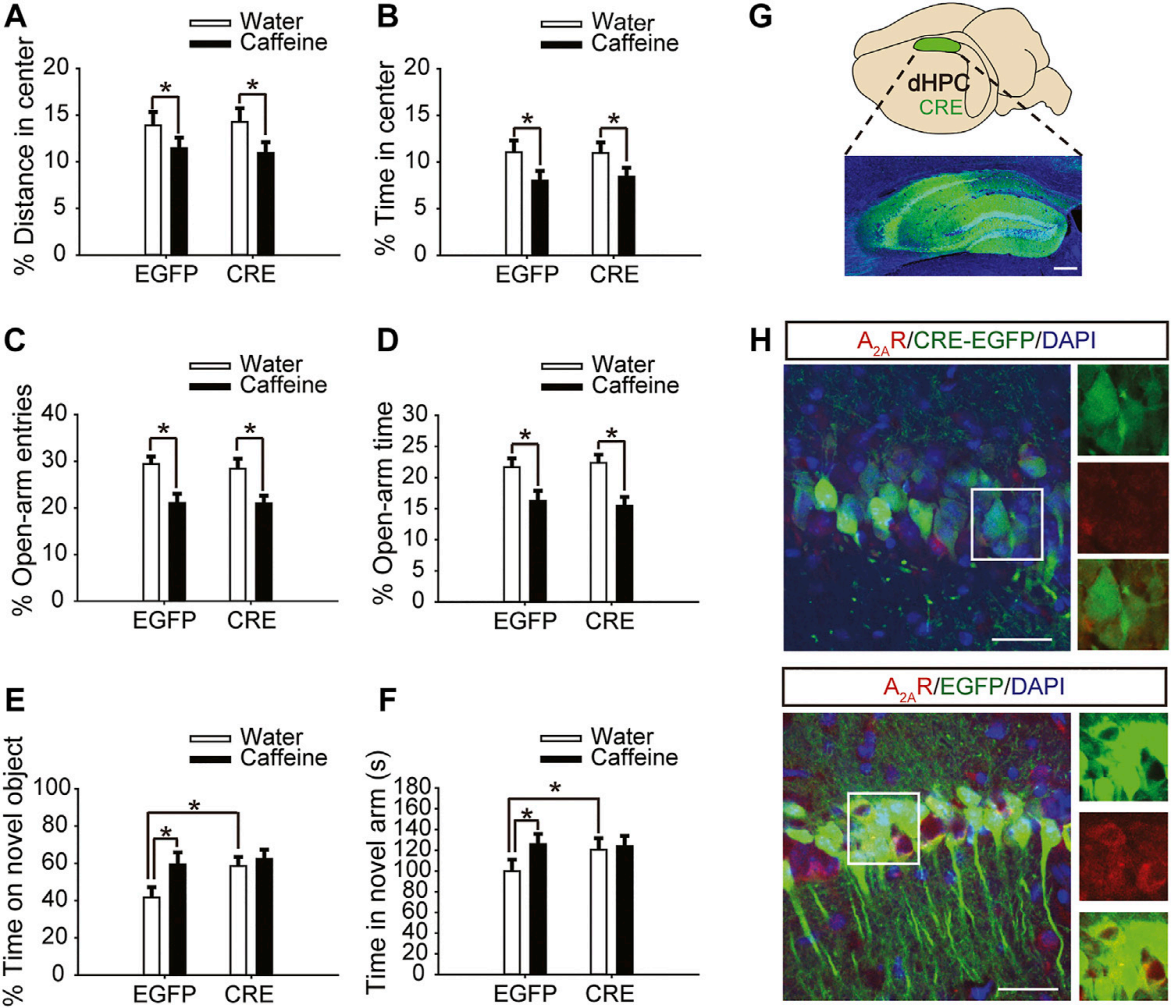
Selective expression of pAAV-CRE in the dHPC triggers memory enhancement. **(A,B)** Behavior of the four groups of mice in the open-field test. Caffeine decreased the time in the center **(B)** and distance in center **(A)** in CRE and EGFP mice. **(C,D)** Behavior of the four groups of mice in the Elevated Plus Maze (EPM) test. Caffeine decreased the percentage of open-arm entries **(C)** and the percentage of time spent in the open arms **(D)** in CRE and EGFP mice. **(E)** Behavior of the four groups of mice in the Novel Object Recognition (NOR) test. The time spent exploring a novel object was increased by caffeine only in EGFP mice during the testing trial. **(F)** Behavior of the four groups of mice in the Y-maze test. The time spent in the novel arm was increased by caffeine only in EGFP mice during the retrieval phase. **(G)** Selected expression of AAV-CRE in the dHPC (scale bar = 500 μm). **(H)** Images of hippocampal brain sections obtained from mice injected with AAV-CRE or AAV-EGFP (scale bar = 30 μm). The level of A_2A_R (red) was lower in CRE-positive cells (upper panel) than in cells transfected with pAAV-EGFP (lower panel). Data are presented as the mean ± SEM; n = 7 mice per group; *p < .05, two-way ANOVA, Bonferroni post hoc t-test.

Six weeks after the injection of pAAV-syn-EGFP-2A-CRE virus in the dHPC of A_2A_^flox/flox^ mice (Figure 2G), the A_2A_R level was significantly reduced (Figure 2H). Then, after drinking caffeine for 3 weeks, mice showed reduced center distance (Figure 2A, for caffeine, F_(1,28)_ = 7.473, *p* < .01; for CRE, F_(1,28)_ = 17.453, *p* = .413; for caffeine × CRE interaction, F_(3,28)_ = 32.913, *p* = .302) and center time (Figure 2B, for caffeine, F_(1,28)_ = 15.417, *p* < .01; for CRE, F_(1,28)_ = 32.348, *p* = .235; for caffeine × CRE interaction, F_(3,28)_ = 42.374, *p* = .328) in the open-field test, and had no significant effect on the total distance (Supplementary Figure S1B, for caffeine, F_(1,28)_ = .042, *p* = 0.839; for CRE, F_(1,28)_ = 1.817, *p* = .190; for caffeine × CRE interaction, F_(3,28)_ = 0.767, *p* = .389). At the same time, caffeine reduced the number of entries (Figure 2C, for caffeine, F_(1,28)_ = 5.113, *p* < .01; for CRE, F_(1,28)_ = 15.457, *p* = .191; for caffeine × CRE interaction, F_(3,28)_ = 21.479, *p* = .208) and duration of time (Figure 2D, for caffeine, F_(1,28)_ = 3.323, *p* < .01; for CRE, F_(1,28)_ = 16.278, *p* = .058; for caffeine × CRE interaction, F_(3,28)_ = 22.143, *p* = .572) spent in the open arm of the EPM. These results indicate that dHPC A_2A_R knockout did not affect the caffeine-induced anxiety-like behavior in mice.

**FIGURE 3:**
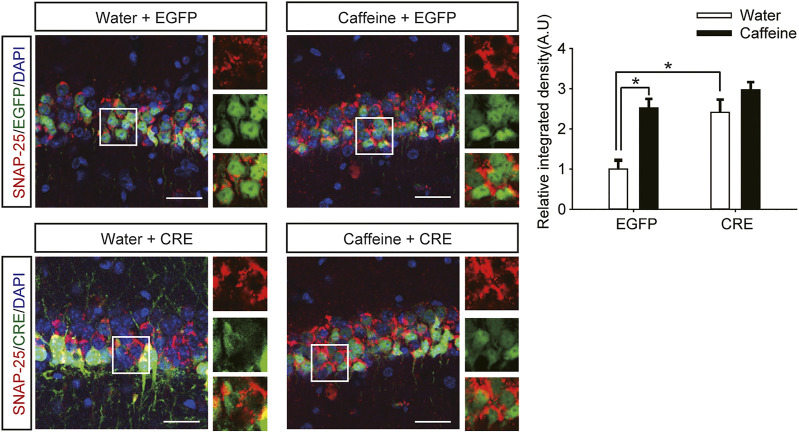
Selective deletion of dHPC A_2A_Rs improves SNAP-25 expression. Caffeine promoted SNAP-25 expression more in mice with the dHPC transfected with pAAV-EGFP than in mice transfected with pAAV-CRE. pAAV-CRE transfection also produced an additive effect on SNAP-25 levels independently. Representative images (left) and data (right) are presented as the mean ± SEM; *n* = 27 fields per group (3 fields per section, three sections per mouse, three mice per group). Scale bar = 30 μm.

However, after inactivation of dorsal hippocampal A_2A_Rs, there was no significant difference in behavior in the NOR test (Figure 2E, for caffeine, F_(1,28)_ = 1.413, *p* < .01; for CRE, F_(1,28)_ = 19.413, *p* < .01; for caffeine × CRE interaction, F_(3,28)_ = 15.439, *p* < .01) or Y-maze test (Figure 2F, for caffeine, F_(1,28)_ = 17.435, *p* < .01; for CRE, F_(1,28)_ = 10.235, *p* < 0.01; for caffeine × CRE interaction, F_(3,28)_ = 5.636, *p* < .01) between the caffeine-treated group and the control group, indicating that dHPC A_2A_R knockout eliminated the effect of caffeine on memory. Immunofluorescence results showed that the level of SNAP-25 in the pAAV-EGFP group given caffeine (EGFP + caffeine) was significantly higher than that in the group given water (EGFP + water). However, caffeine did not alter the expression of SNAP-25 in the pAAV-CRE group (Figure 3). Interestingly, the NOR test and Y-maze test showed that dorsal hippocampal A_2A_R knockout alone improved memory similar to chronic caffeine consumption (Figures 2E,F), and these behavioral changes were in accordance with the trend observed in SNAP-25 expression (Figure 3). This evidence suggests that the effect of caffeine on memory in mice is derived from its action on dorsal hippocampal A_2A_Rs.

### Ventral Hippocampal A_2A_R Knockout Prevented Caffeine-Induced Anxiety

**FIGURE 4:**
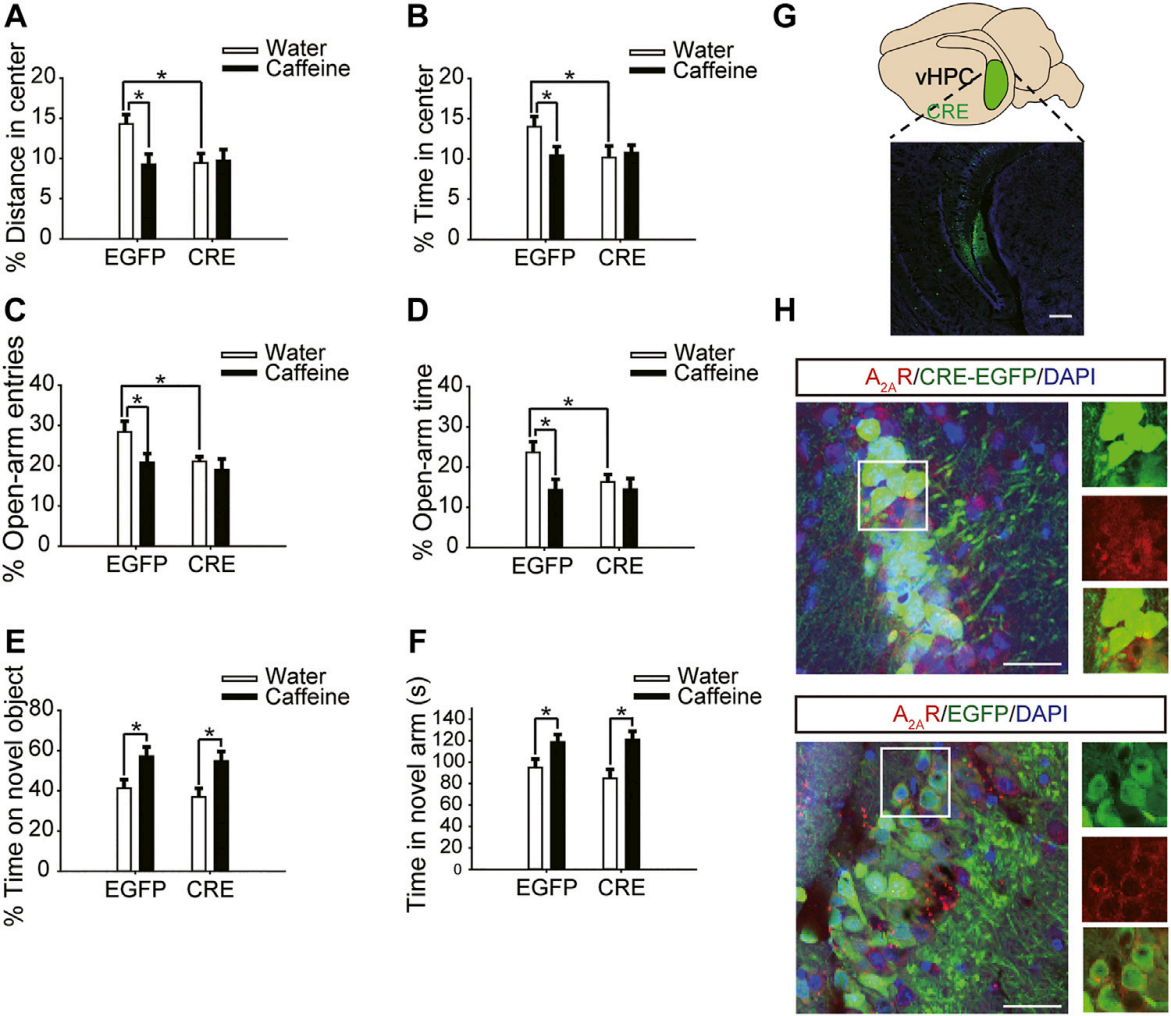
Selective deletion of vHPC A_2A_Rs triggers anxiety-like behaviors. **(A,B)** Behavior of the four groups of mice in the open-field test. Caffeine induced anxiogenic behavior with decreased time in the center **(B)** and distance in the center **(A)** only in EGFP mice. **(C,D)** Behavior of the four groups of mice in the Elevated Plus Maze (EPM) test. Caffeine decreased the percentage of open-arm entries **(C)** and the percentage of time spent in the open arms **(D)** only in EGFP mice. **(E)** Behavior of the four groups of mice in the Novel Object Recognition (NOR) test. The time spent exploring a novel object was increased by caffeine in EGFP and CRE mice during the testing trial. **(F)** Behavior of the four groups of mice in the Y-maze test. The time spent in the novel arm was increased by caffeine in EGFP and CRE mice during the retrieval phase. **(G)** Expression of AAV-CRE or AAV-EGFP in the vHPC (scale bar = 500 μm). Data are presented as the mean ± SEM; *n* = 7 mice per group; *p < .05, two-way ANOVA, Bonferroni post hoc t-test.

**FIGURE 5:**
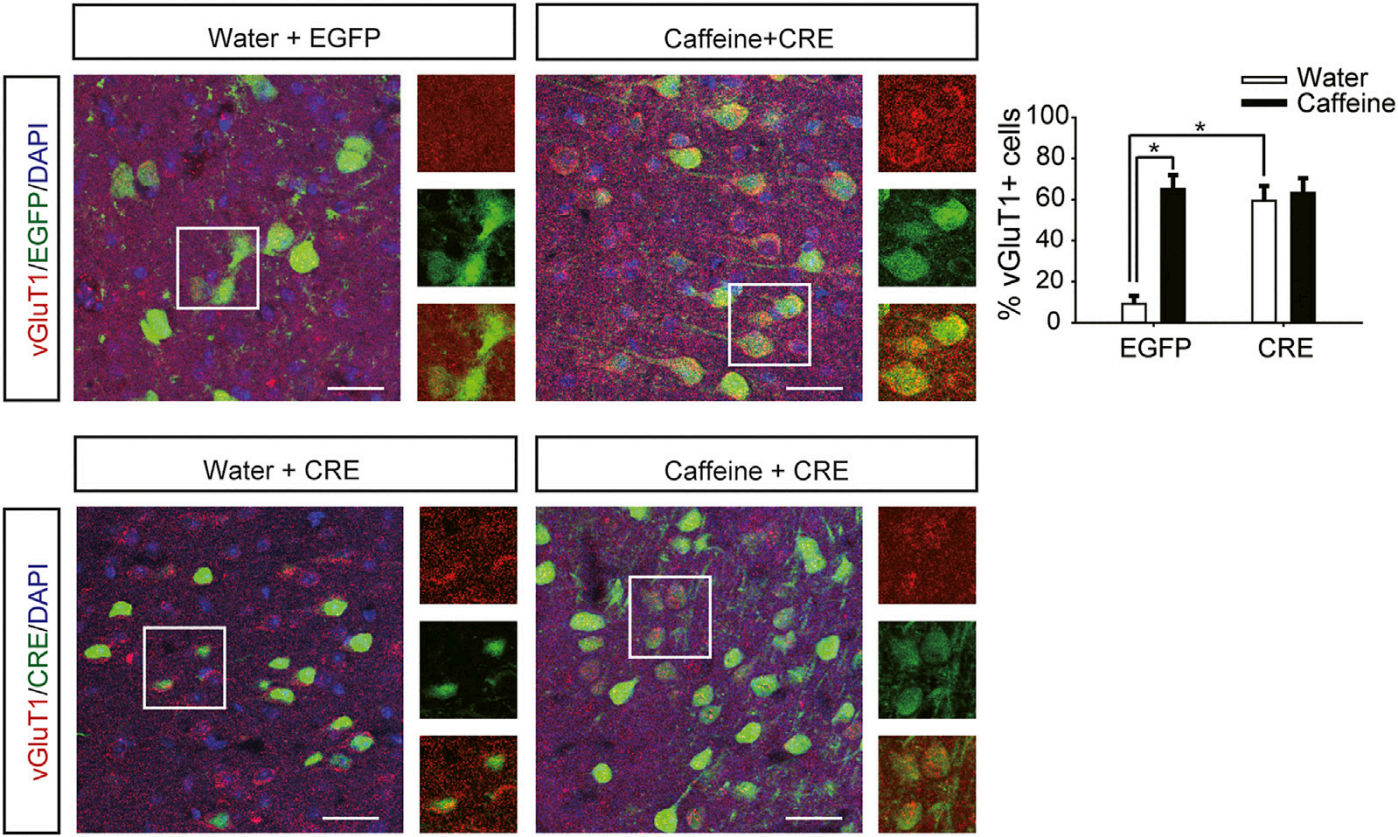
Selective deletion of vHPC A_2A_Rs increases vGluT1 expression. Caffeine markedly induced vGluT1 expression in mice transfected with pAAV-EGFP but not in mice transfected with pAAV-CRE. pAAV-CRE transfection increased vGluT1 levels in mice exposed to water alone as well. Representative images (left) and Data (right) are presented as the mean ± SEM; *n* = 27 fields per group (3 fields per section, three sections per mouse, three mice per group). Scale bar = 30 μm.

After expression of pAAV-syn-EGFP-2A-CRE in the vHPC (Figure 4G), chronic caffeine consumption for 3 weeks had no significant effect on behavior in the open-field test or EPM test (Figures 4A–D), indicating that inactivation of vHPC A_2A_Rs abolished the anxiogenic effect of caffeine. At the same time, mice in the pAAV-CRE group (CRE + water) showed greater anxiety-like behavior than pAAV-EGFP mice (EGFP + water) in the open-field test (Figure 4A, for caffeine, F_(1,28)_ = 15.445, *p* < .01; for CRE, F_(1,28)_ = 17.609, *p* < .01; for caffeine × CRE interaction, F_(3,28)_ = 5.235, *p* < .01; Figure 4B, for caffeine, F_(1,28)_ = 7.143, *p* < .01; for CRE, F_(1,28)_ = 2.439, *p* < .01; for caffeine × CRE interaction, F_(3,28)_ = 8.833, *p* < .01) and EPM test (Figure 4C, for caffeine, F_(1,28)_ = 11.415, *p* < .01; for CRE, F_(1,28)_ = 11.961, *p* < .01; for caffeine × CRE interaction, F_(3,28)_ = 9.675, *p* < .01; Figure 4D, for caffeine, F_(1,28)_ = 21.435, *p* < .01; for CRE, F_(1,28)_ = 19.495, *p* < .01; for caffeine × CRE interaction, F_(3,28)_ = 5.195, *p* < .01), and had no significant effect on the total distance in the open-field test (SupplementaryFigure S1C, for caffeine, F_(1,28)_ = 0.108, *p* = .746; for CRE, F_(1,28)_ = 1.838, *p* = .188; for caffeine × CRE interaction, F_(3,28)_ = 1.367, *p* = .252). The levels of vGLuT1 in the four groups of mice (Figure 5) were consistent with the trend in behavior. Thus, these findings indicate that the anxiogenic effect of chronic caffeine consumption stems from its inhibition of ventral hippocampal A_2A_Rs.

Caffeine in drinking water still increased the time spent exploring a novel object (Figure 4E, for caffeine, F_(1,28)_ = 3.239, *p* < .01; for CRE, F_(1,28)_ = 13.319, *p* = .279; for caffeine × CRE interaction, F_(3,28)_ = 21.173, *p* = .064) and the exploration time of the new arm (Figure 4F, for caffeine, F_(1,28)_ = 7.631, *p* < .01; for CRE, F_(1,28)_ = 23.659, *p* = .288; for caffeine × CRE interaction, F_(3,28)_ = 31.849, *p* = 0.233) in both the pAAV-CRE and pAAV-EGFP mice, indicating that the ablation of ventral hippocampal A_2A_Rs did not affect the memory-enhancing effect of caffeine.

### Brain Region-specific Activation of dHPC/vHPC A_2A_Rs Reversed the Regulation of Caffeine on Memory and Anxiety

**FIGURE 6:**
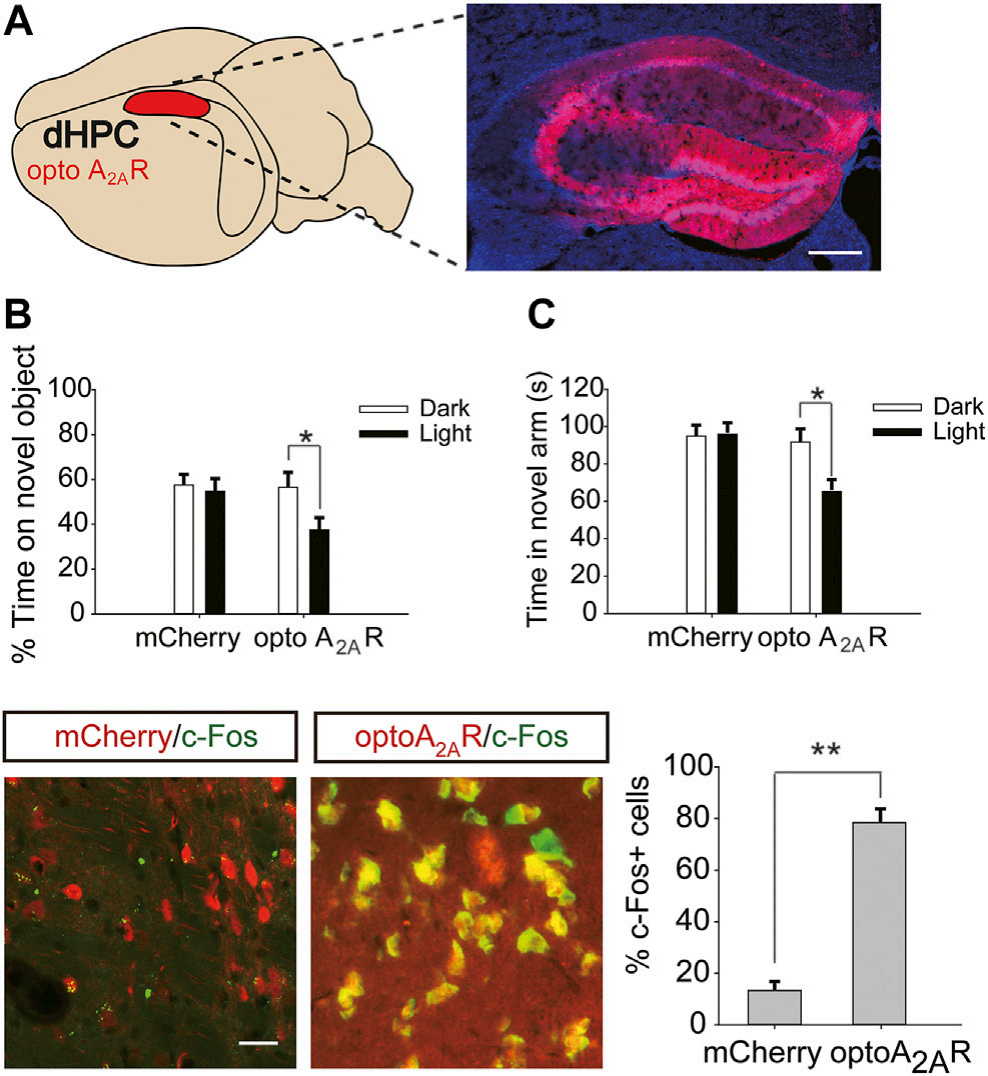
Targeted expression and light activation of optoA_2A_R in the dHPC prevents the memory improvement caused by caffeine consumption. **(A)** Selected expression of AAV-optoA_2A_R or AAV-mCherry in the dHPC (Scale bar = 500 μm). **(B)** Behavior of mice in the Novel Object Recognition (NOR) test (*n* = 7 mice per group). **C** Behavior of the four groups of mice in the Y-maze test (*n* = 7 mice per group). Light stimulation of opto-A_2A_R in the dHPC increased the time spent exploring a novel object during the testing trial (B; *p < .05 using a two-way ANOVA followed by Bonferroni post hoc t-test) and the time spent in the novel arm during the retrieval phase (C; *p < .05 using a two-way ANOVA followed by Bonferroni post hoc t-test). **(D)** Light stimulation for 5 min induced c-Fos expression in optoA_2A_R-positive cells but not in cells transfected with AAV-mCherry. Representative images (left) and data (right) are presented. *n* = 27 fields per group (3 fields per section, three sections per mouse, three mice per group); Scale bar = 50 μm; **p < .001, Student’s t-test comparing optoA2AR with mCherry. Data are presented as the mean ± SEM.

**FIGURE 7:**
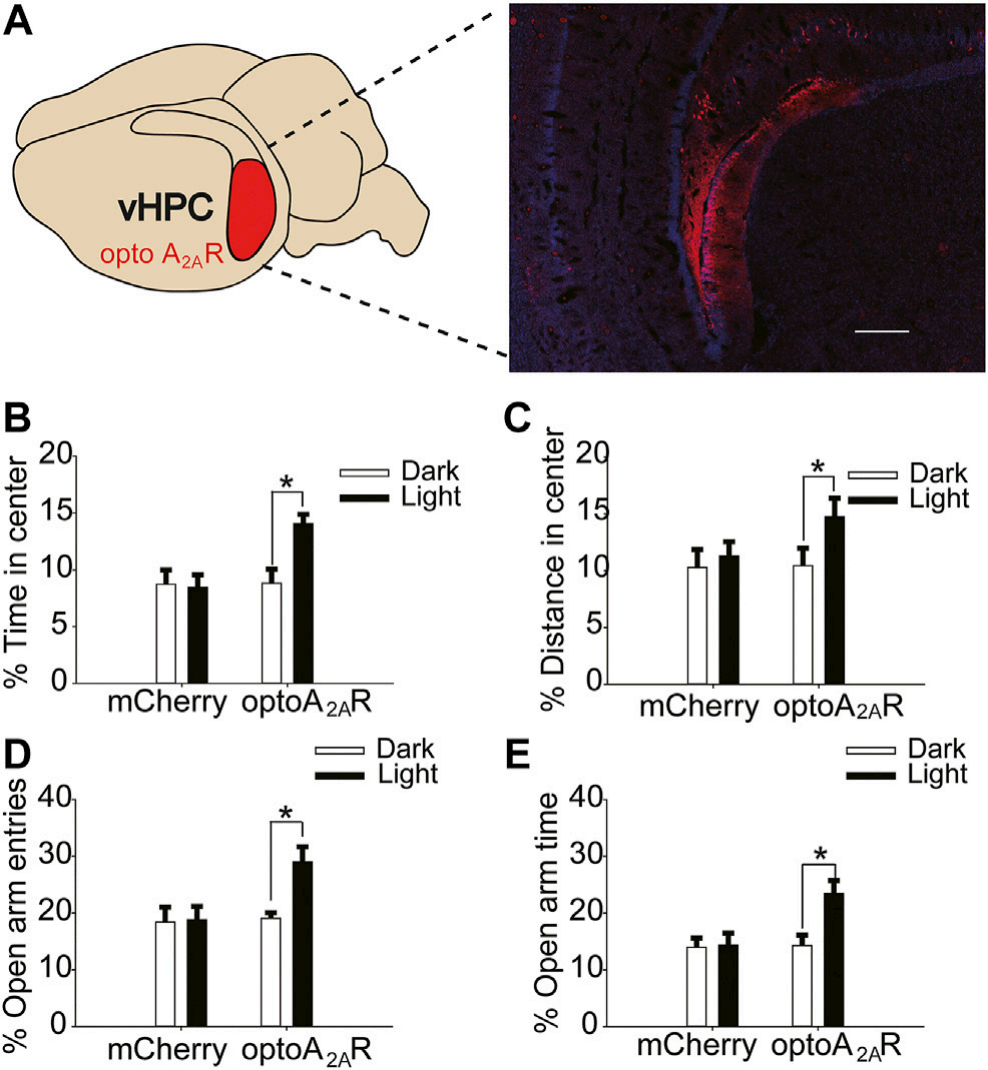
Targeted expression and light activation of optoA_2A_R in the vHPC alleviates caffeine-induced anxiety. **(A)** Selected expression of AAV-optoA_2A_R or AAV-mCherry in the vHPC (Scale bar = 500 μm). **(B,C)** Behavior of the four groups of mice in the open-field test (*n* = 7). Light stimulation of opto-A_2A_R in the dHPC increased the percentage of open-arm entries **(B)** and the percentage of time spent in the open arms **(C)**. **(D,E)** Behavior of the four groups of mice in the Elevated Plus Maze (EPM) test (*n* = 7). Light stimulation of opto-A_2A_R in the dHPC increased the percentage of open-arm entries **(D)** and the percentage of time spent in the open arms **(E)**. Data are presented as the mean ± SEM. *p < .01 using two-way ANOVA followed by Bonferroni post hoc t-test.

Six weeks after dHPC/vHPC injection of pAAV-CaMKIIa-optoA_2A_R-mCherry (Figures 6A,7A), mice were exposed to caffeine in drinking water for 3 weeks. Light stimulation for 5 min significantly increased the levels of c-Fos in pAAV-optoA_2A_R mice but not in pAAV-mCherry mice (Figure 6D), indicating that illumination was sufficient to activate the optoA_2A_R signaling pathway.

After optogenetic optoA_2A_R activation in the dHPC, both the time spent exploring novel objects (Figure 6B, for optoA_2A_R virus, F_(1,28)_ = 3.891, *p* = .105; for light stimulation, F_(1,28)_ = 11.391, *p* < .05; for virus × light interaction, F_(3,28)_ = 25.317, *p* = .091) and time spent in the novel arm (Figure 6C for optoA_2A_R virus, F_(1,28)_ = 16.132, *p* = .067; for light stimulation, F_(1,28)_ = 13.639, *p* < .05; for virus × light interaction, F_(3,28)_ = 12.558, *p* = .334) were significantly decreased, indicating that specific activation of dorsal hippocampal A_2A_Rs triggered obvious memory impairments in mice exposed to caffeine and further suggesting that caffeine promotes memory function by inhibiting dorsal hippocampal A_2A_Rs.

In addition, light activation of optoA_2A_R in the vHPC significantly enhanced the proportion of time spent in the center and distance in the center in the open-field test (Figure 7B, for optoA2AR virus, F_(1,28)_ = 3.229, *p* = .255; for light stimulation, F_(1,28)_ = 8.296, *p* < .01; for virus × light interaction, F_(3,28)_ = 26.539, *p* = .539; Figure 7C, for optoA_2A_R virus, F_(1,28)_ = 11.453, *p* = .548; for light stimulation, F_(1,28)_ = 13.231, *p* < .05; for virus × light interaction, F_(1,28)_ = 23.665, *p* = .792), and had no significant effect on the total distance (Figure S 1Dfor optoA2AR virus, F_(1,28)_ = 0.299, *p* = .589; for light interaction, F_(1,28)_ = 0.088, *p* = .769; for virus × light interaction, F_(3,28)_ = 0.246, *p* = .624).Furthermore, the number of open-arm entries and duration of time spent in the open arms in the EPM increased markedly with light activation (Figure 7D, for optoA_2A_R virus, F_(1,28)_ = 21.238, *p* = .459; for light stimulation, F_(1,28)_ = 17.487, *p* < .001; for virus × light interaction, F_(3,28)_ = 28.292, *p* = .428; Figure 7E, for optoA_2A_R virus, F_(1,28)_ = 3.241, *p* = .536; for light stimulation, F_(1,28)_ = 14.549, *p* < .01; for virus × light interaction, F_(3,28)_ = 13.449, *p* = .413). These findings indicate that specific activation of ventral hippocampal A_2A_Rs inhibited the anxiety-like behaviors of mice treated with caffeine and affirm that caffeine-induced anxiety originates from its suppression of ventral hippocampal A_2A_Rs.

## Discussion

In the present study, we demonstrated that chronic caffeine consumption (1 g/L, 3 weeks) increased anxiety-like behavior, as assessed by the open-field test and EPM, and enhanced memory, as reflected in the NOR and Y-maze. Consistent with our results, caffeine has been shown to reverse cognitive impairments in aging and Alzheimer’s disease (AD) (Solfrizzi et al., 2015; Londzin et al., 2021) and lead to depression and anxiety-like behaviors (Richards and Smith, 2015; Mikkelsen et al., 2017). Acute caffeine consumption is widely used to counteract mood and performance impairments associated with sleep loss (Irwin et al., 2020). To eliminate the acute effects of caffeine while preserving its long-term chronic effects, all behavioral tests were performed after 3-week caffeine treatment. Chronic but not acute treatment with caffeine is considered to induce those behavioral alterations. As caffeine is most widely consumed all over the world, our exploration of chronic caffeine consumption is more meaningful. Moreover, caffeine seemed to have no effects on anxiety and memory in our total A_2A_R knockout mice. Statistical analysis also showed the strong interactions between caffeine and A_2A_R function, which suggests that caffeine functions through the antagonism of A_2A_Rs. The main targets for caffeine in the brain are the inhibitory A_1_R and the facilitatory A_2A_R (Fredholm et al., 2005). Which is different from anti-inflammatory effect of A_1_R against noxious brain conditions (Martins et al., 2015), A_2A_R may play a more important role in memory and anxiety.

Many evidence suggests that pathological brain conditions associated with memory impairment are accompanied by a local increase of the extracellular levels of adenosine (Chen et al., 2013) and an up-regulation and aberrant signaling of the brain A_2A_R (Cunha and Agostinho, 2010; Chen et al., 2013). Thus, A_2A_R blockade could attenuate the impairment of brain function (Cunha, 2016) and memory function in particular (Cunha and Agostinho, 2010). However, whether A_2A_R blockade increases learn and memory in healthy animals remains contentious. Several groups reported that A_2A_R blockade and caffeine did not increase memory performance in control rodents, whereas stressed mice displayed increased memory performance upon caffeine consumption or upon blocking A_2A_R (Prediger et al., 2005; Kaster et al., 2015; Laurent et al., 2016; Carvalho et al., 2019). But genetic KO studies have shown that inactivation of A_2A_R is sufficient to improve memory in healthy animals (Zhou et al., 2009; Wei et al., 2011). The mechanism by which genetic inactivation of A_2A_Rs strengthens memory is not clear. loss of A_2A_Rs may impact cortical function through neuronal networks such as basal ganglia loop (Zhou et al., 2009) or potentiate striatal dopaminergic signaling via D_2_Rs to produce the memory enhancement (Wei et al., 2011).

While studies have shown that intraperitoneal injection of the A_2A_R agonist CGS21680 produces strong anxiety-like behavior (El Yacoubi et al., 2000), in our study, the A_2A_R knockout mice showed greater anxiety and enhanced memory compared to wild-type littermates. Considering that A_2A_R is widely distributed in the brain, the overall effect of drinking caffeine is likely be a superposition of its antagonism towards A_2A_R in multiple brain regions. Therefore, A_2A_R in a particular brain subregion may be responsible for caffeine’s effects on anxiety-like and memory-related behaviors.

Another major advance provided by this study is the dissociation of the roles of the ventral and dorsal hippocampal A_2A_ receptors in caffeine’s effects on anxiety-like and memory-related behavioral measures, respectively, which are also consistent with the reported roles of the dorsal and ventral hippocampus more generally (Fanselow and Dong, 2010; McHugh et al., 2011; Schumacher et al., 2018). We discovered that knocking out dorsal hippocampal A_2A_Rs blocked the memory-enhancing effects of caffeine without affecting its anxiogenic effects, whereas knocking out ventral hippocampal A_2A_Rs did not affect the memory-enhancing effects of caffeine but blocked its anxiogenic effects. In addition, there were baseline differences in the behaviors of site-specific A_2A_R knockout mice, and statistical analysis confirmed caffeine-A_2A_R interactions. These results indicate that caffeine modulates memory by inhibiting dorsal hippocampal A_2A_R and modulates anxiety by acting through ventral hippocampal A_2A_R.

To confirm the memory-enhancing effects dHPC A_2A_R knockout, we assessed the level of SNAP-25. Our study showed that inactivation of dHPC A_2A_R upregulated the density of synaptic proteins, consistent with a previous study that showed that activation of hippocampal A_2A_R is sufficient to attenuate synaptic plasticity and further impair memory (Li et al., 2015). In recent years, synaptic density and synaptic connections in the hippocampus have been associated with learning and memory (Benito and Barco, 2010; Latina et al., 2017), and the overexpression of adenosine receptors revealed a hippocampal LTD-to-LTP shift to impair synaptic plasticity (Temido-Ferreira et al., 2018). Thus, chronic caffeine consumption may affect synaptic function to enhance memory by inhibiting dHPC A_2A_Rs. However, the specific mechanism remains to be further confirmed.

One of the most important hypotheses of anxiety disorder is inhibition/excitation imbalance (Colic et al., 2018). As vGluT1 is a glutamatergic-selective marker and labels excitatory glutamatergic neurons (Zheng et al., 2015; Martineau et al., 2017), upregulation of vGluT1 reflects excessive excitation and inhibition/excitation imbalance in vHPC to some extent. It is also consistent with the greater anxiety-like behavior induced by caffeine and dHPC adenosine A_2A_ receptor knockout. It is unclear whether caffeine regulates anxiety through the alteration of vGluT1, but the activation of adenosine A_2A_ receptor has been reported to reduce GluT and glutamate uptake in cultured astrocytes and gliosomes (Matos et al., 2012). Therefore, the upregulation of vGluT1 may underlie the effect of caffeine on anxiety via inhibition of vHPC adenosine A_2A_ receptor.

As a supplement to the brain region-specific knockout of adenosine A_2A_ receptor, an optoA_2A_R approach to mimic endogenous A_2A_R signaling was used. Due to the specific construct, overexpression of optoA_2A_R did not generate baseline effects and caffeine could not react with optoA_2A_R too. After 3-weeks antagonism by caffeine, endogenous A_2A_R signaling was inhibited, optogenetic activation of optoA_2A_R captured the physiological function of the native A_2A_R. Light activation of dHPC adenosine A_2A_ receptors reversed the behavioral alterations caused by caffeine, leading to impaired memory, while light activation of vHPC adenosine A_2A_ receptors decreased caffeine-induced anxiety, further confirming the dissociation between the roles of the ventral and dorsal hippocampal A_2A_ receptors in caffeine’s effects. Considering that we examined the behavioral effects of withdrawal from a chronic regime of caffeine administration, caffeine may induce compensatory effects in response to long-term drug exposure. The instant but significant effect of optoA_2A_R suggests the specificity of A_2A_R signaling rather than the compensatory effects.

Since adeno-associated viral vectors driven by either the synapsin- (syn-) or CaMKIIa promoter were employed, our results suggested that the effects of caffeine may result from specific inhibition of neuronal adenosine A_2A_ receptors, consistent with previous studies on the role of neuronal adenosine A_2A_ receptor in cognition (Kaster et al., 2015; Viana da Silva et al., 2016; Temido-Ferreira et al., 2018). The prominent cortical connectivity of the dorsal hippocampus and the projection to the anterior cingulated cortices are involved in memory processing (Jones and Wilson, 2005; Lavenex et al., 2006). The CA1 and subiculum of the ventral hippocampus share massive bidirectional connectivity with amygdala nuclei, which plays a key role in control (Pitkanen et al., 2000; Liu and Carter, 2018). The neuronal adenosine A_2A_ receptors in those projections may underlie the distinct regulation of adenosine A_2A_ receptors in the dHPC and vHPC. No method was designed in this research to confirm the mechanism, and further research is needed to clarify the mechanisms of neuronal A_2A_R-mediated regulation and modulation of caffeine.

## Conclusion

In the present study, we blocked and reversed the effects of caffeine by specifically inactivating and activating dHPC/vHPC adenosine A_2A_ receptors. For the first time, caffeine was demonstrated to affect memory by inhibiting dorsal hippocampal adenosine A_2A_ receptors and affect anxiety by inhibiting ventral hippocampal adenosine A_2A_ receptors, explaining how caffeine triggers anxiety while enhancing memory. Our results may help to understand the mechanisms of anxiety and memory and provide an experimental basis for making better use of caffeine while avoiding its side effects.

## Data Availability

The original contributions presented in the study are included in the article/Supplementary Material, further inquiries can be directed to the corresponding authors.
