# Supplementary figures and images for: Caffeine Functions by Inhibiting Dorsal and Ventral Hippocampal Adenosine 2A Receptors to Modulate Memory and Anxiety, Respectively
Source: Front Pharmacol. 2022 Feb 2;13:807330. doi: 10.3389/fphar.2022.807330 (PMC8847668; doi:10.3389/fphar.2022.807330)

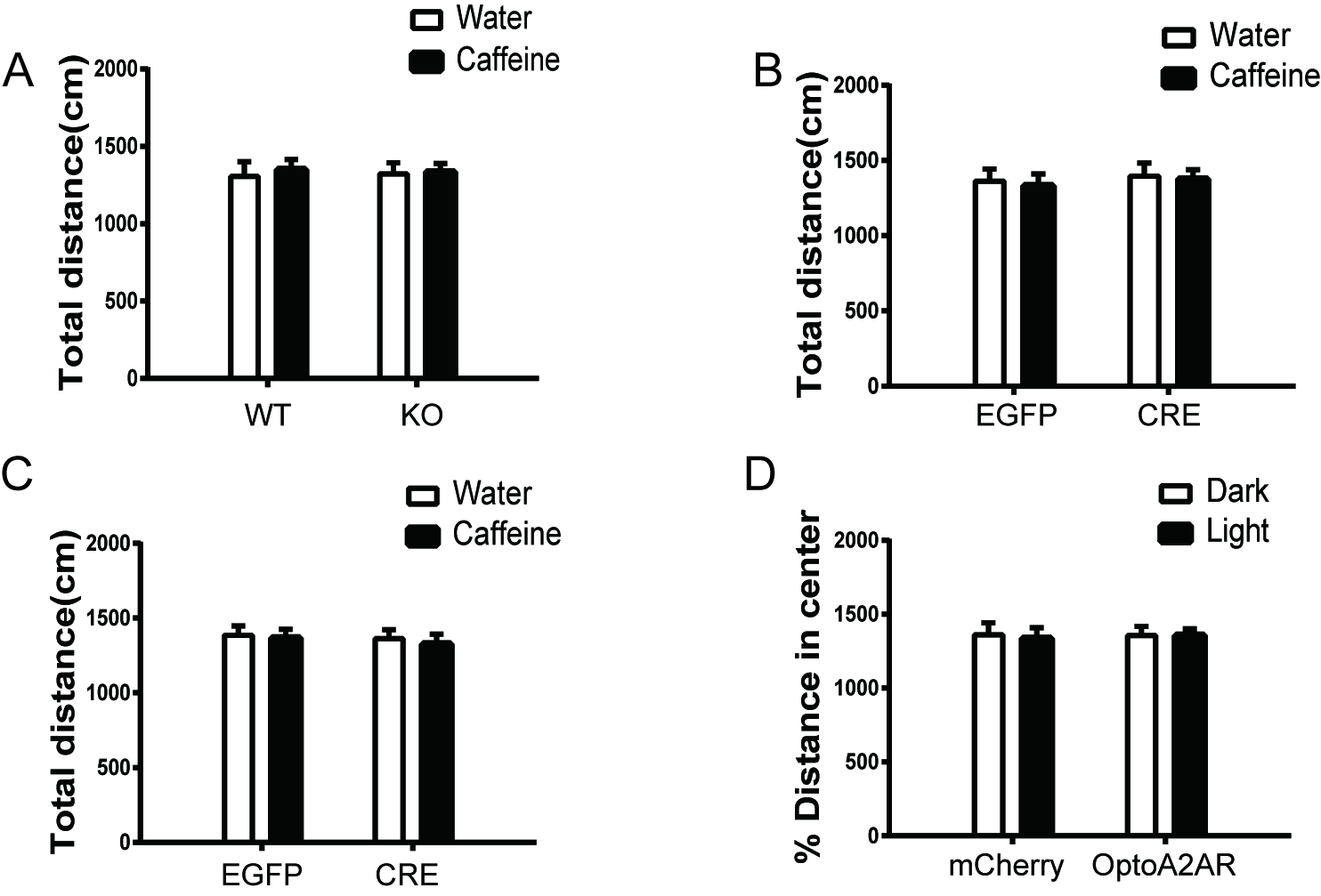

Supplement: Supplementary file 1 [file Image1.TIF]
